# Time Course Analysis of Transcriptome in Human Myometrium Depending on Labor Duration and Correlating With Postpartum Blood Loss

**DOI:** 10.3389/fgene.2022.812105

**Published:** 2022-06-28

**Authors:** Lina Chen, Yihong Luo, Yunshan Chen, Lele Wang, Xiaodi Wang, Guozheng Zhang, Kaiyuan Ji, Huishu Liu

**Affiliations:** Guangzhou Key Laboratory of Maternal-Fetal Medicine, Guangzhou Women and Children’s Medical Center, Guangzhou Medical University, Guangzhou, China

**Keywords:** labor duration, cervical dilation, postpartum blood loss, myometrium, transcriptome, expression regulation

## Abstract

The maintenance of coordinated powerful episodic contractions of the uterus is the crucial factor for normal labor. The uterine contractility is gradually enhanced with the progression of labor, which is related to the gene expression of the myometrium. Competing endogenous RNA (ceRNA) can also regulate the gene expression. To better understand the role of ceRNA network in labor, transcriptome sequencing was performed on the myometrium of 17 parturients at different labor durations (0–24 h). From this, expression levels of mRNA, long non-coding RNA (lncRNA), circular RNA (circRNA), and microRNA (miRNA) were correlated with labor duration. Then, targeting relationships between mRNAs, lncRNAs, circRNAs, and miRNAs were predicted, and the ceRNA regulatory network was established. The mRNA expression patterns associated with cervical dilation and postpartum bleeding were further investigated. This analysis identified 932 RNAs positively correlated with labor duration (859 mRNAs, 28 lncRNAs, and 45 circRNAs) and 153 RNAs negatively correlated with labor duration (122 mRNAs, 28 lncRNAs, and 3 miRNAs). These mRNAs were involved in protein metabolism, transport, and cytoskeleton functions. According to the targeting relationship among these ceRNAs and mRNAs, a ceRNA network consisting of 3 miRNAs, 72 mRNAs, 2 circRNAs, and 1 lncRNA was established. In addition, two mRNA expression patterns were established using time-series analysis of mRNA expression in different phases of cervical dilation. A ceRNA network analysis for blood loss was performed; postpartum bleeding was closely related to inflammatory response, angiogenesis, and hemostasis. This study identified human myometrial transcriptome and established the ceRNA regulatory network depending on labor duration and highlighted the dynamic changes that occur at ceRNAs during parturition, which need to be considered more in the future to better understand how changes in gene expression are relevant to functional changes in human myometrium at labor.

## Introduction

During labor, the myometrium undergoes a series of sustained and powerful contractions to deliver the baby, a process that produces biochemical and structural changes in the myometrium. The first stage of labor contains the latent phase and the active phase. The latent phase is characterized by slow cervical dilation and varies in duration. As labor progresses, the cervix dilates more rapidly, commonly commences from 4 cm dilation, and the intensity of uterine contractions increases which leads into the active phase, with regular and strong uterine contractions (Friedman, 1955; Krapohl et al., 1970; Liao et al., 2005). Changes in uterine myometrial contractility are underpinned by complex and highly regulated processes, cell structure, and signaling of the myometrium (Li et al., 2021), such as an increase in contractile proteins and changes in glycolytic and oxidative enzymes (Breuiller-Fouche et al., 2006; Wray et al., 2019).

RNA sequencing (RNA-seq) is currently one of the most commonly used methods for high-throughput analysis of gene expression. In previous studies, the complete transcriptome profiles of human myometrium in both quiescent and active states have been sequenced (Ackerman et al., 2021; Chan et al., 2014). Differential analyses of the myometrial transcriptome profiles at different states of cervical dilatation and fetal membrane rupture (ROM) emphasized that a single state of the myometrial transcriptome was unable to represent the physiological dynamic process of labor, and that the different stages of labor are needed to be characterized (Lai et al., 2021). The gene expression in the myometrium may also be influenced by the duration of labor. However, the issue has received little attention and the evidence is inadequate.

In addition, mRNA expression alone is insufficient to elucidate the effects of labor duration on gene expression, as the translation of functional proteins is prone to post-transcriptional regulation. Non-coding RNAs (ncRNAs), including lncRNA, circRNA, and miRNA, play an important role in the post-transcriptional regulation of mRNA. miRNAs function by binding to target mRNA, thus degrading mRNA or inhibiting its translation (Ambros, 2004). lncRNA and circRNA can competitively bind to miRNA through their miRNA responsive elements, thereby effectively controlling the subsequent post-transcriptional regulation of miRNA, reducing the inhibition of miRNA on mRNA expression, and acting as competing endogenous RNA (ceRNA) (Kopp et al., 2018; Zhang et al., 2020). ncRNA has key roles in the governance of myometrial contractility. Previous studies have shown that miRNAs, such as miR-200 family and miR-199a/214 cluster, participated in the hormonal regulation of myometrial quiescence and contractility during pregnancy and labor through the regulation of inflammation- and contraction-associated gene expression (Renthal et al., 2013). lncRNA has been considered to be the most frequent, prevalent, and abundant novel class of human genes (Guttman et al., 2012). Illumina^®^ microarray of myometrium identified 1,692 lncRNAs, of which 13 were differentially expressed (Romero et al., 2014). Despite the importance of ncRNA in the myometrium during labor, there is little information about ncRNA at different phases of labor and lack of regulatory analysis of ncRNA and mRNA.

Cesarean section increases the amount of bleeding after delivery, compared to vaginal delivery (Misme et al., 2016). The most common cause of bleeding is uterine atony (Breathnach et al., 2009), and the main mechanism for preventing excessive bleeding is uterine myometrial contraction and thrombosis. Myometrial muscle fibers stretch in different directions during uterine contractions, squeezing the large blood vessels and therefore controlling bleeding. Pregnancy is primarily a hypercoagulable state to prevent postpartum hemorrhage, and defects in coagulation pathways may also lead to excessive bleeding (Oyelese et al., 2010). The function of the uterine myometrium is closely related to bleeding after delivery. Studies on the correlation between the functions of the myometrium and bleeding at the transcriptome level would help to find the molecular mechanisms of the myometrium in regulating bleeding after delivery.

This study aims to provide a comprehensive workflow and analysis of the expression of ncRNA and mRNA in human term gestation pregnancy myometrium. A correlation analysis approach was used to screen RNAs associated with labor duration or postpartum bleeding, and an analysis of their functions and regulations was performed to reveal the biochemical and structural dynamics of the myometrium in labor, so as to identify effective targets for real-time monitoring of labor.

## Materials and Methods

### Subjects and Tissue Collection

A total of 17 lower uterine segment samples were collected from singleton, nulliparous women undergoing cesarean deliveries at different labor durations, including 2 non-labor (labor duration of 0 h) and 15 spontaneous term in labor (labor duration of 5–24 h). The tissue samples in this study overlapped with those in our previous publication (Chen et al., 2021), a study analyzing mRNA differences between non-laboring (n = 10) and laboring (n = 10) myometrial samples, in which mRNA data from 2 randomly selected non-laboring and 10 laboring samples were used. Five additional myometrial samples at different labor durations were collected, forming a cohort with a labor duration of 0–24 h. The clinical details of patients gathered using clinical phenotype and statistical testing are presented in Supplementary Table S1. This research was approved by the Ethics Committee of Guangzhou Women and Children Medical Center (No. 201915401), and the informed consent form was signed by every participant.

The participants underwent a cesarean section for indications of breech, fetal distress, or cephalopelvic disproportion, with no pregnancy (pre-labor) complications, placenta previa, or uterine fibroids. Labor was defined as regular palpable contractions (assessed using cardiotocography) and cervical dilation (assessed by digital examination). Each patient’s labor duration was documented from labor start to cesarean section. The starting point was determined using cardiotocography and by digital examination after self-reporting regular contractions. Postpartum blood loss included bleeding from fetal delivery to 2 h postoperation. The quantitative postpartum bleeding was calculated by measuring the blood in the aspirator during operation and the blood in the blood-soaked gauze or nursing pad postoperation (Wilcox et al., 1959). Vaginal speculum examination was used for determining fetal membrane rupture status (ROM).

Myometrial tissue samples were obtained from the lower uterine segment during cesarean section after delivery of the fetus and placenta. Tissue samples were immediately washed with phosphate-buffered saline (Sigma) to reduce the amount of blood, and the attached decidua and adipose tissue were removed using surgical scissors and then dissected into pieces of approximately 100 mg and immersed in RNAlater solution (Sigma) to be snap frozen in liquid nitrogen and stored at −80°C.

### Total RNA Extraction

For each sample, total RNAs were extracted from a minimum of 60 mg myometrium tissues, and detailed steps were as stated in our previous study (Chen et al., 2021). All samples (including those from previous study) were sequenced for mRNA and non-coding RNA.

### mRNA and Long Non-coding RNA Library Construction and Sequencing

Methods for library construction are as stated in our previous study (Chen et al., 2021). The qualified libraries were pair end sequenced on the BGISEQ-500 System (BGI-Shenzhen, China). HISAT2 (v2.0.4) and RSEM (v1.2.12) were used to map and count the reads of mRNA and lncRNA with the reference of human genome (H. sapiens, GRCh38) and transcriptome (Ensembl, release 84) (Kim et al., 2015; Li et al., 2011). All the datasets presented in this study were deposited in Genome Sequence Archive (GSA) repository with accession number PRJCA009585.

### miRNA Library Construction and Sequencing

Total RNAs were separated using polyacrylamide gel electrophoresis (PAGE). The 15% TBE-urea gel was compounded and pre-runned for 15–30 min at 200 V, RNA ladder and total RNA sample were mixed with gel loading dye, respectively, and then heated at 65°C for 5 min. The entire RNA ladder and total RNA sample were loaded onto the gel, and the gel was run at 200 V for 1 h. Small RNA regions corresponding to the 18–30 nt bands in the marker lane (14–30 ssRNA Ladder Marker, TAKARA) were excised and recovered. Then the small RNAs were ligated to adenylated 3’ adapters which were annealed to unique molecular identifiers (UMI), followed by the ligation of 5’ adapters. The adapter-ligated small RNAs were transcribed into cDNA and subsequently enriched using PCR. The target fragments of 110–13 bp were selected using agarose gel electrophoresis and purified using a QIAquick Gel Extraction Kit (QIAGEN, Valencia, CA). The library was checked for the distribution of fragments size using the Agilent 2100 bioanalyzer and quantified using real-time quantitative PCR (qPCR) (TaqMan Probe). The final ligated PCR products were sequenced using the BGISEQ-500 platform (BGI-Shenzhen, China). The cleaned reads were mapped to the miRBase with Bowtie2 (Langmead et al., 2009), and cmsearch (Nawrocki et al., 2013) was performed for Rfam mapping. The miRDeep2 software was used to predict novel miRNA by exploring the secondary structure (Friedländer et al., 2008). All the datasets presented in this study were deposited in Genome Sequence Archive (GSA) repository with accession number PRJCA009585.

### circRNA Library Construction and Sequencing

Total RNAs were treated with DNase I and a Ribo-off rRNA Depletion Kit (Vazyme, Inc.) to degrade DNA and ribosomal RNA, respectively. Linear RNA was removed using RNase R (Epicentre, lnc). Purification was performed using Agencourt RNAClean XP magnetic beads. A tailing mix and RNA index adapters were added to perform end repair. The PCR products were denatured and circularized using the splint oligo sequence. Single-strand circular DNA was formatted as the final library. The library was checked for the distribution of fragments size using the Agilent 2100 bioanalyzer and quantified using BMG microplate reader (OMEGA). Finally, the qualified libraries were pair end sequenced on the BGISEQ-500 (BGI-Shenzhen, China). The software CIRI and find_circ is used to predict circRNA (Gao et al., 2015; Memczak et al., 2013). All the datasets presented in this study were deposited in Genome Sequence Archive (GSA) repository with accession number PRJCA009585.

### Identification of Labor Duration or Blood Loss–Correlated RNAs

In RNA-seq data analysis, fragments per kilo base per million mapped reads (FPKM) and reads per million mapped reads (RPM) are two kinds of normalized expression units to remove technical biases such as the depth of sequencing and gene length. FPKM considers the sequencing depth and gene length for normalization and is suitable for paired-end RNA-seq protocols where gene length fluctuates greatly, such as mRNA, lncRNA, and circRNA sequencing (Conesa et al., 2016; Trapnell et al., 2010). RPM considers the sequencing depth but not the transcript length normalization and is suitable for sequencing protocols where reads are generated irrespective of gene length, such as miRNA-seq, as miRNA lengths are typically between 20 and 24 bp (Campbell et al., 2015). In this study, the expression levels of mRNAs, lncRNAs, and circRNAs were presented as FPKM values; the expression levels of miRNAs were presented as RPM values. RNAs with extremely low abundance (average FPKM/RPM of 17 samples <1) were excluded.

The correlation between RNA expression levels and labor duration or blood loss was calculated using the “cor.test” function of Pearson correlation in R (v4.1.1), with *p*-value < 0.01 as the threshold for statistical significance. The “pheatmap” and “ggplot2” R packages were used to draw heat map (SCR_016418 and SCR_014601; https://scicrunch.org/resources). A scatter plot of RNA expression values was drawn using Microsoft Excel.

### Gene Function Annotation

Gene ontology (GO) and signaling pathway analysis were conducted on the significantly correlated mRNAs using DAVID v6.8 and KOBAS-i online tools, respectively (Bu et al., 2021; Huang et al., 2009), to annotate the biological processes (BP), cellular component (CC), molecular function (MF), and the signaling pathways. GO enrichment analysis and the network construction were performed using ClueGO plug-in of Cytoscape v3.7.2 software (Shannon et al., 2003). Fisher’s exact test is adopted to measure the gene enrichment in annotation terms; a *p*-value < 0.05 was considered to be significantly GO enriched. A corrected *p*-value < 0.05, corrected by Benjamini–Hochberg method, was considered to be significantly enriched.

### circRNA/lncRNA-miRNA-mRNA ceRNA Network Construction

The three correlated miRNAs were selected as the hub components, and the interaction relationships between miRNAs and mRNAs were predicted using microT-CDS (Paraskevopoulou et al., 2013), with a threshold of 0.8. miRNAs that interacted with circRNAs were predicted using the circBank Database (Liu et al., 2019). miRNAs that interacted with lncRNAs were predicted using LncBase v2 of Experimental module (Paraskevopoulou et al., 2016). The three miRNAs targeting mRNAs, circRNAs, and lncRNAs were then selected by overlapping with correlated RNAs. The circRNA/lncRNA–miRNA–mRNA ceRNA network was visualized using Cytoscape v3.7.2 software based on the targeting relationships.

### mRNA Expression Profile Time Series Clustering

The degree of cervical dilation was divided into three phases: cervical dilation = 0 cm (non-labor), cervical dilation <4 cm (the latent phase of the first stage of labor), and cervical dilation ≥4 cm (the active phase of the first stage of labor) (Friedman, 1996). All identified mRNAs were clustered using Short Time-Series Expression Miner (STEM) v1.3.13 (Ernst et al., 2006). Expression profiles of mRNAs were clustered based on FPKM value changes over different cervical dilation phases; the maximum number of model profiles was set to 50, and the maximum unit change in model profiles between time points was set to two. A corrected *p*-value < 0.01 was considered to be significantly enriched. The mRNA relative expression values can be exported from the STEM. The gene expression at the first time point was set to zero, representing the baseline of gene expression.

## Results

### Clinical Characteristics of the Participants

Our study recruited 17 primigravida women with singleton pregnancies. The median and range of labor duration, cervical dilation, and postpartum blood loss were 12 (0–24) h, 3 (0–10) cm, and 330 (250–620) ml, respectively. The indications for caesarean section included the following: fetal distress (n = 9), breech (n = 2), and cephalopelvic disproportion (failure to progress) (n = 6). The clinical details of patients were gathered by the clinician.

### Identification of Labor Duration–Correlated mRNAs

**FIGURE 1:**
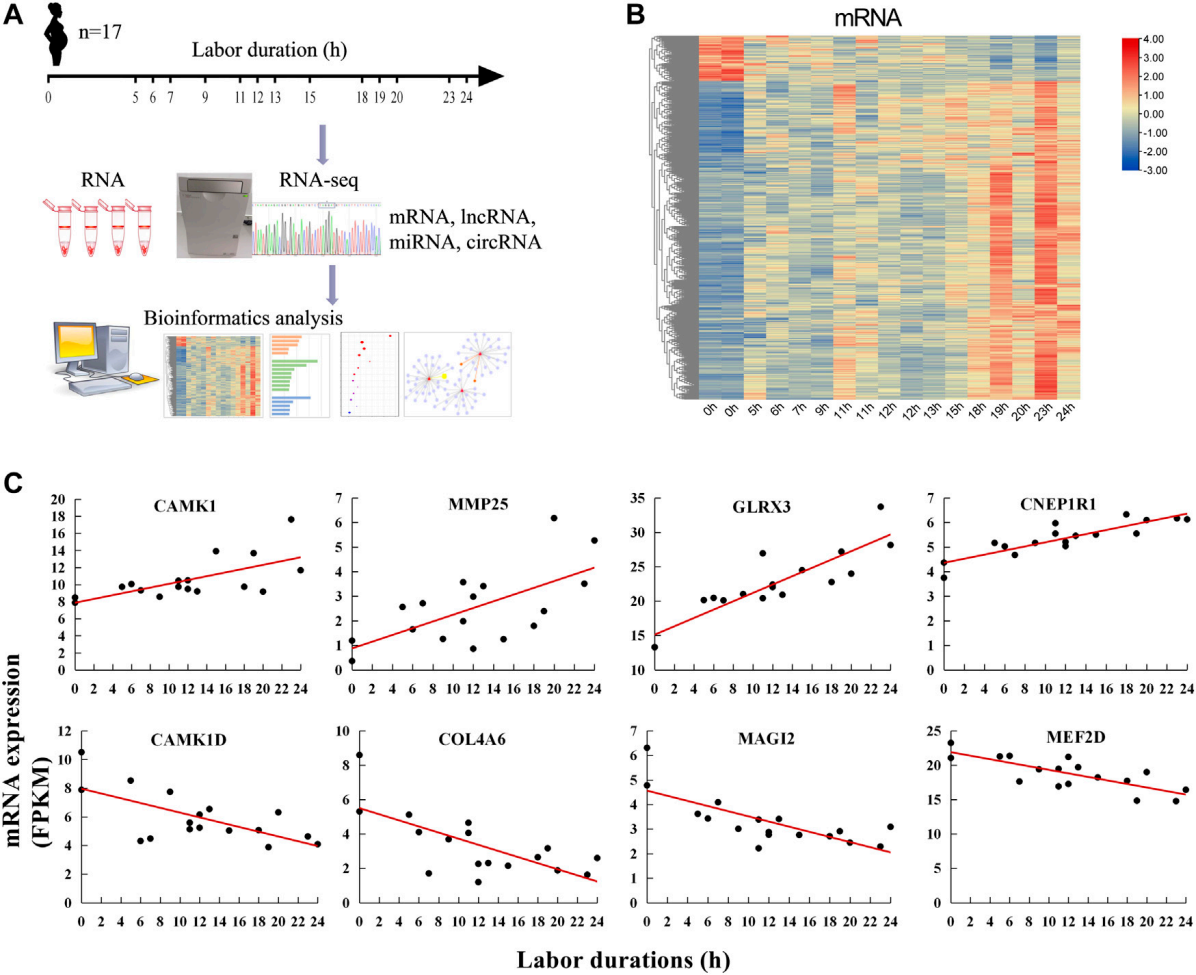
Labor duration–correlated mRNAs expression. **(A)** Overall study design and workflow. **(B)** Heat map of expression of labor duration–correlated mRNAs. Data associated with this figure can be found in Supplementary Table S2. **(C)** The expression trend of mRNAs at different labor durations.

Myometria were collected from 17 parturients undergoing different phases of labor. To identify genes whose expression levels were gradually up- or down-regulated following the duration of labor, the correlation between mRNA expression and labor duration was calculated. The results showed that 859 mRNAs were positively correlated with labor duration and 122 mRNAs were negatively correlated (Figure 1B, Supplementary Table S2). There were some known labor-associated players including (but not limited to) mRNA-encoding proteins involved in the breakdown of extracellular matrix (matrix metalloproteinase 25, MMP25) (Flores-Pliego et al., 2015), cell extracellular matrix interactions (collagens type IV alpha 6 COL4A6) (Shchuka et al., 2020), and calcium signaling regulation (calcium/calmodulin-dependent protein kinase I, CAMK1, and ID, CAMK1D) (Papandreou et al., 2004). The top two mRNAs with the highest positive correlation coefficient (R) with labor duration were glutaredoxin-3 (GLRX3) and CTD nuclear envelope phosphatase 1 regulatory subunit 1 (CNEP1R1). While the top two mRNAs negatively correlated with labor duration were membrane-associated guanylate kinase, WW And PDZ domain containing 2 (MAGI2) and myocyte enhancer factor 2D (MEF2D) (Figure 1C). These findings suggested that substantial transcriptional changes occurred in the myometrium during labor and gene expression changes depended on labor duration.

**FIGURE 2:**
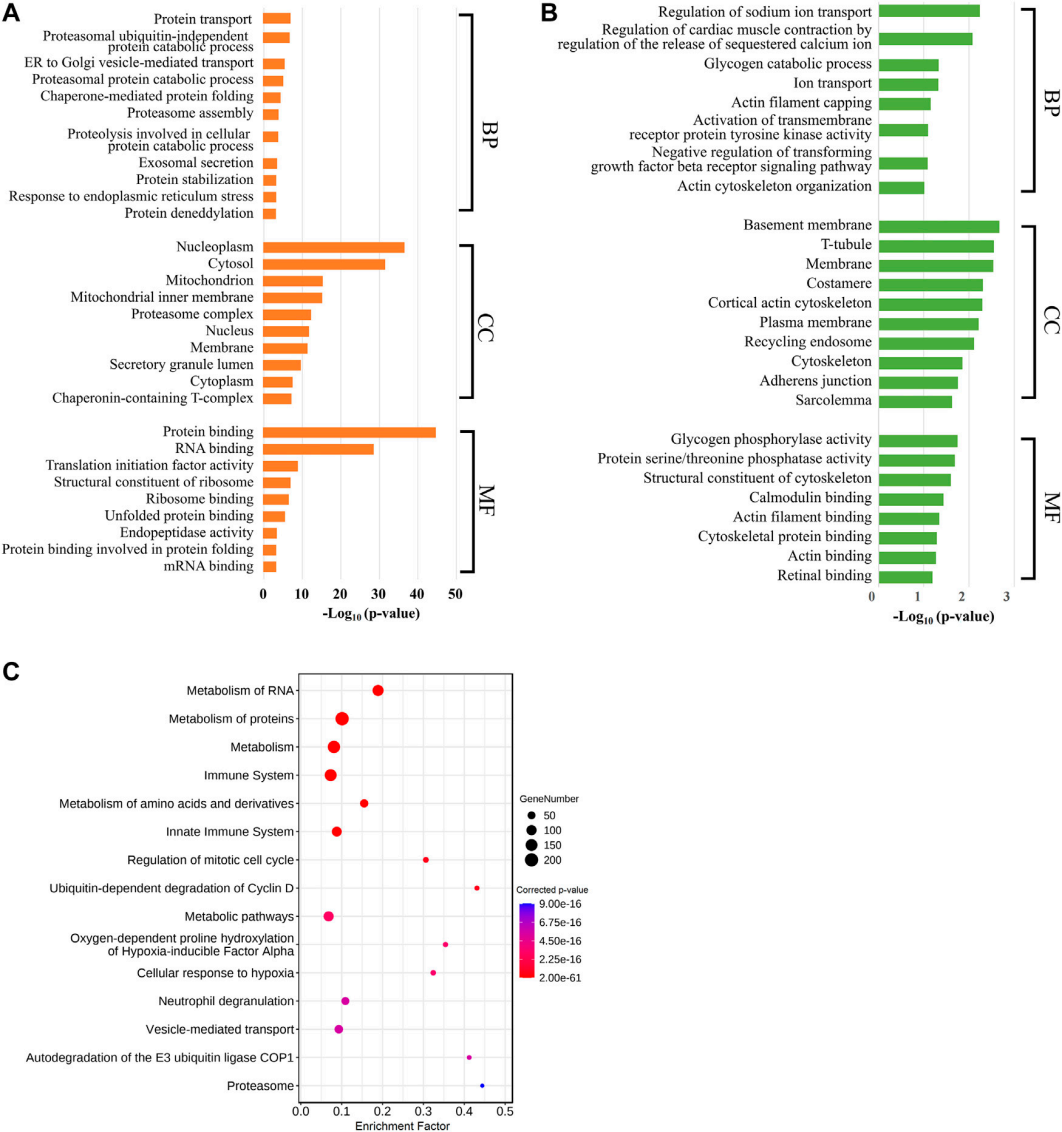
Labor duration–correlated mRNAs function annotation. **(A)** Bar diagram of partial GO term clusters of positively correlated labor duration mRNAs. **(B)** Bar diagram of partial GO term clusters of negatively correlated labor duration mRNAs. **(C)** Bubble diagram of significantly enriched pathways for labor duration–correlated mRNAs.

To better comprehend the functions of these labor duration–correlated mRNAs, we carried out GO and KEGG pathway enrichment analyses. The results of GO enrichment in positively correlated mRNAs revealed that the most enriched BP GO terms were associated with protein metabolic process, especially protein ubiquitination. In addition, vesicle-mediated transport and exosomal secretion were also significantly enriched. The CC GO enriched terms showed that these genes were mostly involved in the composition of nucleoplasm and cytoplasm. In MF GO terms, these genes were significantly enriched for the binding of protein, ribosome, and RNA (Figure 2A, Supplementary Table S3). Meanwhile, in GO analysis of mRNAs negatively correlated with labor duration, the mRNAs were mainly concentrated in the BP of ion transport and actin regulation, and in CC constituting cytoskeleton components such as T-tubule, costamere, cortical actin cytoskeleton, adherens junction, and sarcolemma, the MF terms were enriched in calmodulin and actin binding (Figure 2B, Supplementary Table S4). The two groups of mRNAs with expression levels positively or negatively correlated with labor duration were enriched in different GO terms, indicating that biological processes such as material transportation, metabolism, and cell structure were dynamically changing during labor. The KEGG enrichment analysis of the labor duration–correlated mRNAs showed that multiple pathways were associated with metabolism, immune, and hypoxia response (Figure 2C, Supplementary Table S5), indicating that the gene expression was constantly being regulated during labor, as detected.

### Labor Duration–Related ceRNA Regulatory Network Construction

ncRNAs including lncRNA, circRNA, and miRNA play important roles in post-transcriptional regulation, which ultimately affects mRNA translation (Panni et al., 2020). To explore the regulatory effect of ncRNAs on mRNA during labor, we identified lncRNA, circRNA, and miRNA expression levels that were significantly correlated with labor duration using high-throughput sequencing and correlation analysis. The lncRNA/circRNA-miRNA-mRNA regulatory network was further established.

**FIGURE 3:**
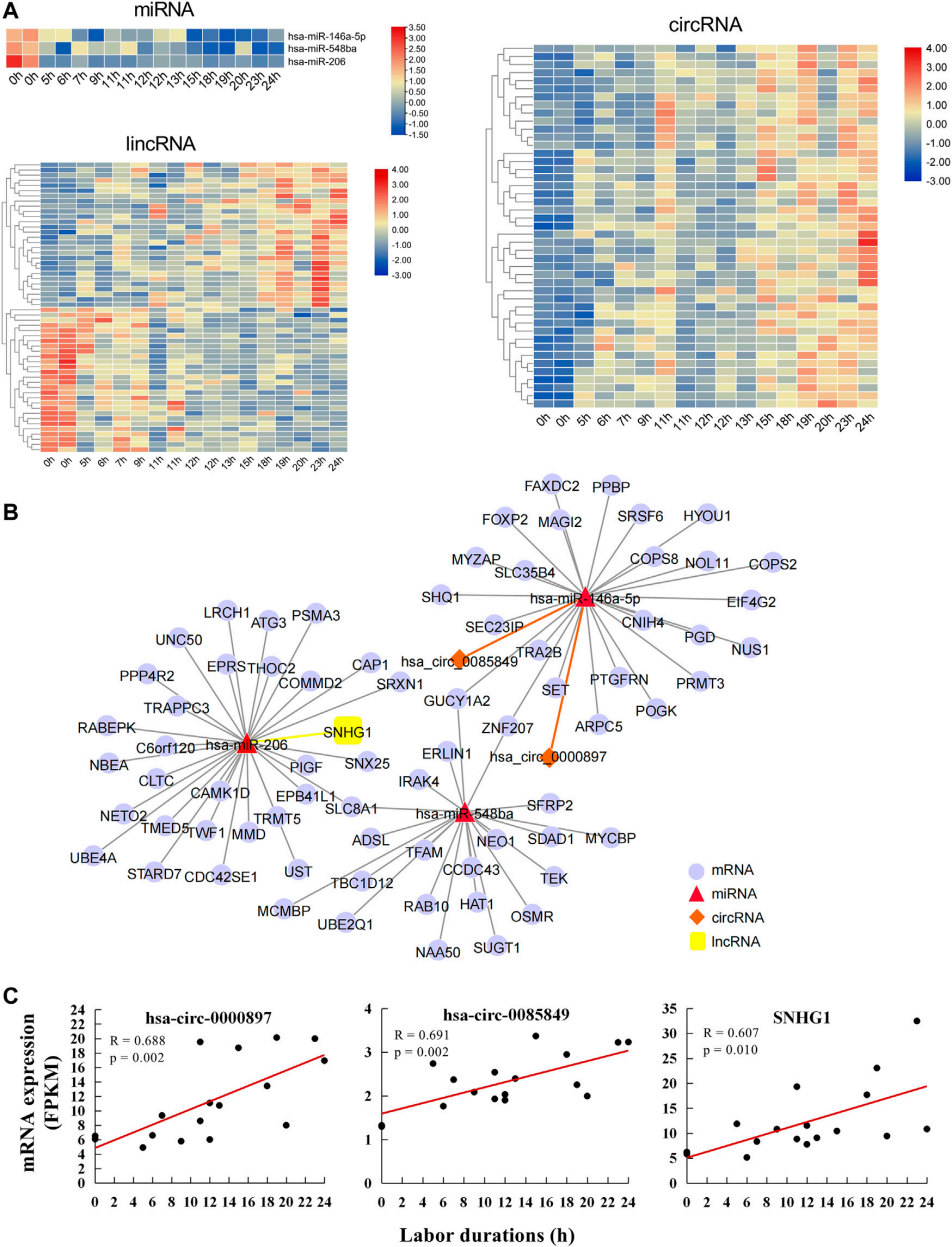
Labor duration–correlated circRNA/lncRNA–miRNA–mRNA ceRNA network. **(A)** Heat map of expression of labor duration correlated miRNAs, lncRNAs, and circRNAs. Data associated with this figure can be found in Supplementary Table S6. **(B)** Labor duration correlated ceRNA network. **(C)** The expression trend of ncRNAs in ceRNA network at different labor durations.

A total of 56 lncRNAs correlated with labor duration (28 positively correlated and 28 negatively correlated), 45 circRNAs positively correlated with labor duration, and three miRNAs negatively correlated with labor duration (Figure 3A, Supplementary Table S6). lncRNA, circRNA, and mRNA all have response elements to bind miRNA directly, which enables them to communicate with and co-regulate each other by competing for binding to the shared miRNAs. Based on the ceRNA theory (Panni et al., 2020), the three miRNAs were defined as the core nodes of the regulatory network, and their target lncRNA, circRNA, and mRNA were predicted through the database. These predicted targets then overlapped with the identified labor duration–correlated lncRNAs, circRNAs, and mRNAs, which were regarded as elements of the ceRNA regulatory network. Finally, there were 75 interaction pairs predicted between three miRNAs and 72 mRNAs, two interaction pairs predicted between one miRNA (hsa-miR-146a-5p) and two circRNAs (hsa_circ_0000897 and hsa_circ_0085849), and one interaction pair predicted between miRNA and lncRNA (hsa-miR-206 and SNHG1). lncRNA/circRNA–miRNA–mRNA ceRNA regulatory networks were constructed based on their targeting relationships. There were three mRNAs (SLC8A1, ZNF207, and GUCY1A2) that can be targeted by two miRNAs (Figure 3B), which were important nodes of the network. The two circRNAs and one lncRNA of the regulatory networks were all positively correlated with labor duration (Figure 3C), which might be key regulatory ncRNAs for parturition.

### Time-Series Analysis of mRNA With Cervical Dilation

**FIGURE 4:**
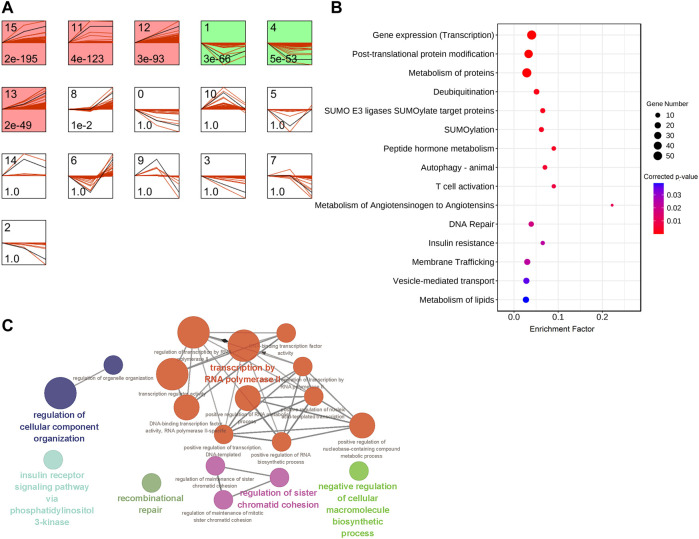
Analysis of mRNAs expression temporal trends and functions in different phases of cervical dilatation. **(A)** STEM analysis of the mRNAs expression temporal trends pattern. The top number indicates the serial number of patterns and the bottom number indicates *p*-value. Colored profiles indicate significant clustered (*p* < 0.01) and the same color represents a similar expression pattern. Colored models meant a statistically significant number of genes were enriched. **(B)** Bubble diagram of partial significantly enriched pathways for mRNAs with more than two-fold change in expression in STEM profile 1. **(C)** ClueGO plot of enriched BP GO annotation for mRNAs with more than two-fold change in expression in STEM profile 1.

Uterine muscle contraction during labor is closely related to cervical dilation. In the latent phase, uterine muscle contractions progress slowly. Then, the speed of dilation accelerates after 4 cm dilation, and when the myometrium reaches an active phase, the contractions become stronger and more regular. In order to identify mRNAs with significant changes in expression between latent and active phases in labor, we analyzed the time-series characteristics of RNA expression in the myometrium during labor with a cutoff of 4 cm cervical dilation. STEM, a tool for the analysis of short-time series gene expression data, was used to cluster and visualize possible changes in the profiles of all 19094 detected mRNAs at three cervical dilation phases: non-labor (n = 2), in labor dilation <4 cm (n = 8), and in labor dilation ≥4 cm (n = 7). There were six significant cluster expression patterns (red and green profiles) in the classified analysis results. In most of the significant profiles, mRNA expressions followed the same trend, either up- or down-regulated, into the active phase. It is worth noting that the mRNA expression in profile 1 did not follow the same trend, but was down-regulated at dilation <4 cm phase followed by returning to the baseline at dilation ≥4 cm phase (Figure 4A). Considering the latent phase (labor dilation <4 cm) which was the beginning of the labor (the first stage of labor), the mRNAs transiently down-regulated at this phase in profile 1 and raised the possibility that some mRNAs might function specifically in triggering labor onset. STEM analysis was performed only on those cases that did not exhibit failure to progress or only on those failed to progress, and the results showed similar mRNA expression temporal trends. Specifically, for profile 1 in Figure 4A, the “up-down” pattern of change in gene expression was also observed in the samples from other two groups (Supplementary Figure S1A,B).

There were 493 mRNAs in profile 1 which were expressed more than two-fold changes in the two phases during labor, and the mRNA relative expression values (compared with group A) were exported from the STEM analysis tool (Supplementary Table S7). Further pathway analyses indicated that mRNAs which specifically down-regulated at dilation <4 cm phase were mainly enriched in transcription, metabolism of proteins and lipids, SUMOylation, and transportation (Figure 4B, Supplementary Table S8). GO enrichment analysis showed these mRNAs were enriched in transcription, chromosome repair, regulation of cellular component organization and macromolecule biosynthetic process, and insulin receptor signaling pathway via phosphatidylinositol 3-kinase (Figure 4C). These findings suggested that metabolic and transcriptional regulations were important for the initiation of labor.

### Identification of Blood Loss–Correlated RNAs

**FIGURE 5:**
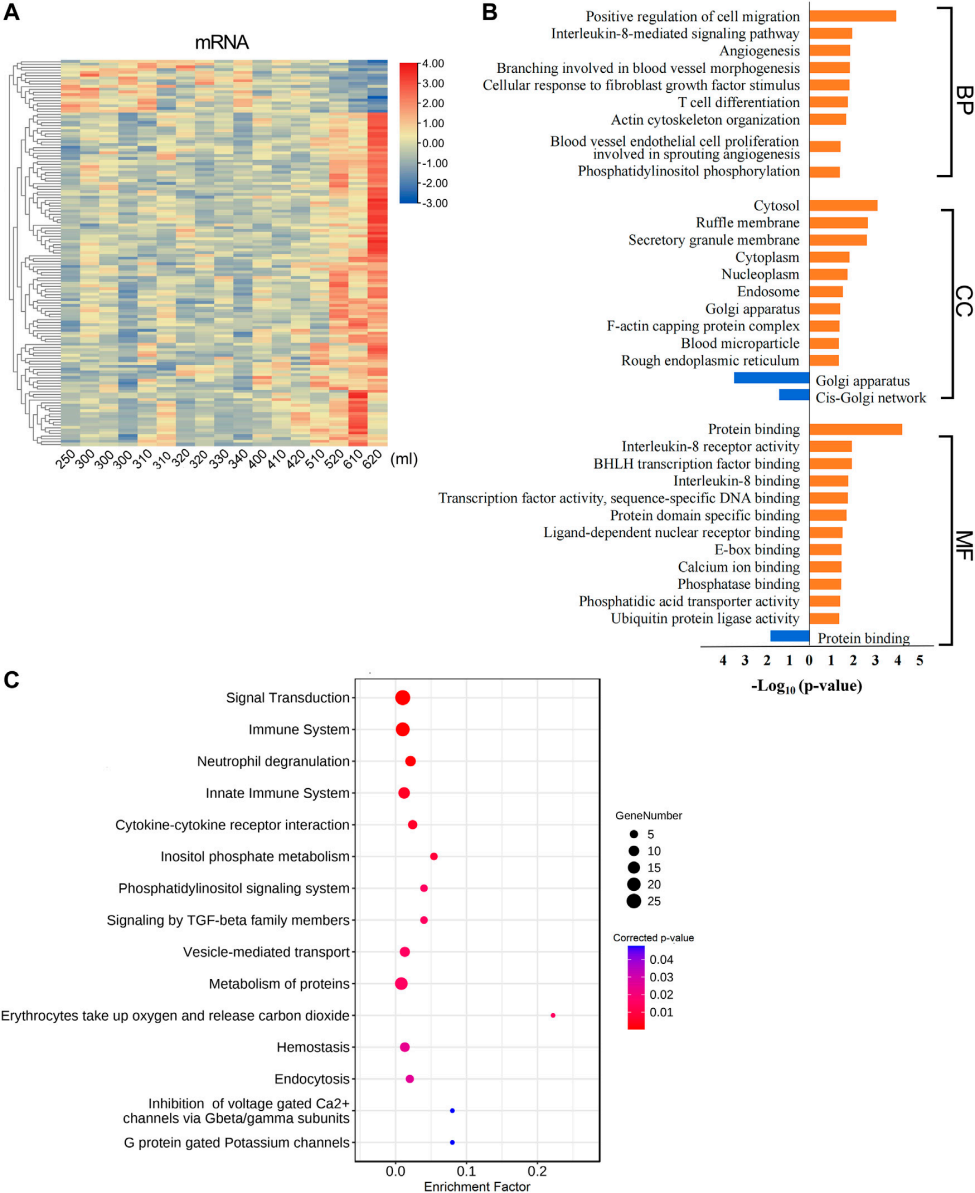
Postpartum bleeding correlated mRNAs function annotation. **(A)** Heat map of expression of postpartum bleeding–correlated mRNAs. Data associated with this figure can be found in Supplementary Table S9. **(B)** Bar diagram of partial GO term clusters of positively (orange) and negatively (blue) correlated postpartum bleeding mRNAs. **(C)** Bubble diagram of significantly enriched pathways for postpartum bleeding–correlated mRNAs.

The total amount of postpartum bleeding was quantified, and the correlation between bleeding volume and RNAs expression was analyzed. A total of 120 mRNAs were positively correlated and 19 mRNAs were negatively correlated with blood loss (Figure 5A, Supplementary Table S9). Functional enrichment analysis of the mRNAs correlated with postpartum bleeding revealed that these genes were associated with multiple coagulation-related functions and pathways. The mRNAs positively correlated with blood loss were enriched in cell migration, cellular response to fibroblast growth factor stimulus, and inflammatory response. CC GO terms focused on blood microparticle and haptoglobin–hemoglobin complex. In MF GO terms, protein binding, interleukin-8 receptor activity, and ubiquitin protein ligase activity were significantly enriched (Figure 5B, Supplementary Table S10). The pathways were enriched in hemostasis, voltage-gated Ca^2+^ channels, and immune system (Figure 5C, Supplementary Table S11). In addition, it was found that there were multiple functions related to immune and inflammatory responses, such as T cell differentiation, chemotaxis, and cytokine–cytokine receptor interaction. The functions of ferrous iron transmembrane transport and magnesium ion binding were also enriched significantly (Figures 5B,C). These results highlighted the important roles of coagulation and contraction in blood loss during labor.

**FIGURE 6:**
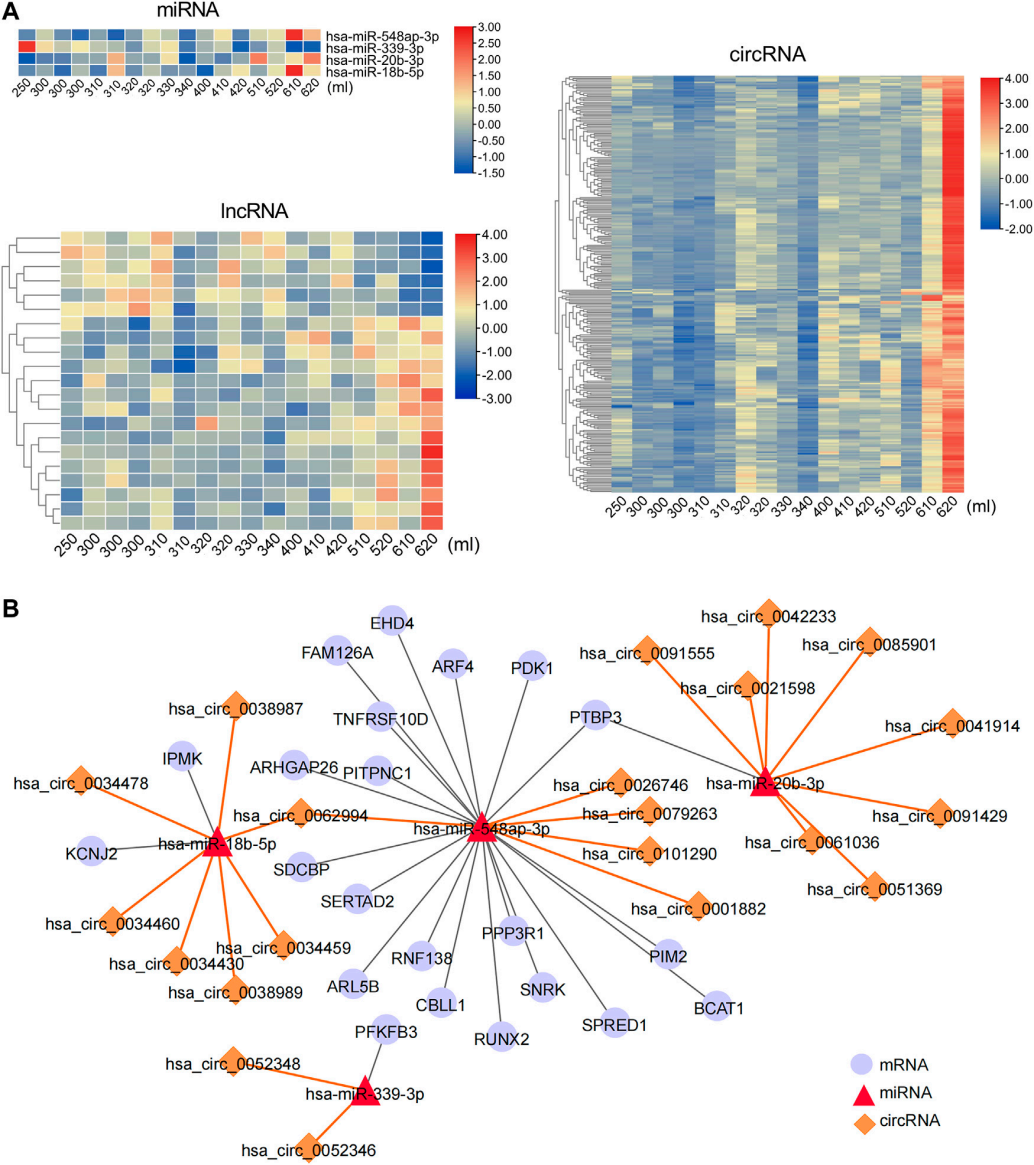
Postpartum bleeding-correlated circRNA/lncRNA–miRNA–mRNA ceRNA network. **(A)** Heat map of expression of Postpartum bleeding-correlated miRNAs, lncRNAs, and circRNAs. Data associated with this figure can be found in Supplementary Table S12. **(B)** Postpartum bleeding–correlated ceRNA network.

To further explore the regulatory effect of ncRNAs on mRNAs related to postpartum blood loss, ncRNAs that correlated with blood loss were identified. A total of 297 ncRNAs were positively correlated with blood loss, including three miRNAs, 15 lncRNAs, and 279 circRNAs. Seven RNAs were negatively correlated with blood loss, including one miRNA and six lncRNAs (Figure 6A, Supplementary Table S12). Based on the predicted interaction relationships of the blood loss–correlated RNAs, a regulatory network consisting of four miRNAs, 22 mRNAs, and 21 circRNAs was constructed. The mRNA PTBP3 and circRNA hsa_circ_0062994 were the nodes of the network targeting two miRNAs, respectively (Figure 6B), suggesting their role as possible key regulators for postpartum blood loss.

## Discussion

Labor, a physiologic and continuous process, is traditionally divided into three stages. The first stage refers to the interval between the onset of labor and full cervical dilatation. In the first stage, the myometrial contraction gradually becomes intense and regular, and the cervix gradually dilates (Liao et al., 2005). In this study, the transcriptome (both mRNA and ncRNA) of the myometrium from different labor durations and cervical dilation was sequenced. It was a time-course analysis that detailed a novel workflow for observing time-dependent changes in myometrial tissue gene expression for ceRNA network analysis during first stage labor over the course of 24 h, and a separate analysis was conducted for cervical dilation and postpartum blood loss status with the same study cohort. Our study presented both mRNA and ncRNA transcriptome data for each study participant, and found postpartum blood loss was correlated with changes in myometrial gene expression. Some genes and pathways closely related to labor durations and cervical dilation may be important targets for regulating myometrial contraction.

A variety of differentially expressed genes between the in labor and not in labor myometrium have been identified in previous studies. The roles of extracellular matrix interaction and calcium signal regulation in the myometrium during labor have been confirmed (Liao et al., 2005). In our analysis, mRNA-encoding proteins known to be involved in functions such as MMP25, COL4A6, CAMK1, and CAMK1D were also found to be associated with labor duration. In correlation analysis, a total of 981 mRNAs were identified to have expression levels significantly varied with the time of labor duration. In STEM analysis for the same samples, there were 493 mRNAs down-regulated in the latent phase and then up-regulated in the active phase, as shown in profile 1 derived from the STEM analysis. Many of these mRNAs were enriched in gene expression–related pathways or biological processes such as RNA degradation and transport, transcription, and protein ubiquitination, indicating that during labor, the genes expressed in the myometrium were constantly being regulated along with the process of labor.

The constant regulation of gene expression in the myometrium during labor resulted in the changes of numerous biological functions. According to the results of functional enrichment analysis of the labor duration–correlated mRNAs, metabolic process was found to be the most prominent enrichment. The myometrium undergoes hypertrophy during pregnancy, storing large amounts of glycogen, lipid, and protein in preparation for labor (Scheepers et al., 2001). The biological processes of glucose and lipid metabolism, which were the main energy supplies, were significantly enriched in mRNAs positively correlated with labor duration. Labor is an energy-intensive process, and an up-regulation of glucose and lipid metabolism can support the intense contraction of the myometrium. Our previous metabolomic profile analysis of myometrium showed that metabolism increased during labor, especially lipolysis and fatty acid oxidation (Qian et al., 2021).

As for protein metabolism, the most significant functions and pathways were protein ubiquitination and deubiquitination, which were enriched by mRNAs positively correlated with labor duration and mRNAs in STEM profile 1, suggesting a potential association to autophagy, as the results showed that the expression of autophagy-related mRNAs also increased during the active phase. Our previous studies showed that autophagy was activated in human myometrium during labor (Wang et al., 2020). Autophagy may serve as protection during transient ischemia and hypoxia caused by uterine contraction (Yan et al., 2013). Studies have shown that there was a complex cross-talk between ubiquitin-proteasome system and autophagy. Protein ubiquitination mediates autophagy and controls the initiation, execution, and termination of autophagy along with deubiquitination (Chen et al., 2019; Shaid et al., 2013). Thus, we speculate that myometrial autophagy may begin to prepare in the latent phase and occur in the active phase. The functions enriched by the mRNAs transiently down-regulated at the latent phase, such as deubiquitination, autophagy, and vesicle-mediated transport, might participate in triggering labor onset.

ceRNA network, which has been proved to be ubiquitous in post-transcriptional regulation of gene expression, interconnects encoding and non-encoding RNAs regulation and works with other cellular and molecular regulation mechanisms (Tay et al., 2014). ceRNA networks were previously reported in carcinogenesis (Ala, 2021), yet it is unclear in the myometrium during labor. The ceRNA network constructed in this study demonstrated the regulatory relationship among different kinds of transcripts correlated with labor duration. We identified three mRNAs that formed the connection points of the whole network; specifically SLC8A1, GUCY1A2, and ZNF207. SLC8A1 encodes sodium/calcium exchanger protein, which contributes to Ca^2+^ transport during excitation–contraction coupling in muscle (Shattock et al., 2015). GUCY1A2 encodes the α2 subunit of soluble guanosine cyclase (sGC), and the sGC-catalyzed production of cyclic guanosine phosphate (cGMP) is involved in the relaxation of smooth muscle in human vas deferens and airways (Britt et al., 2015; Da et al., 2012). ZNF207 encodes kinetochore- and microtubule-binding protein that participates in spindle assembly by blocking ubiquitination and proteasomal degradation of mitotic checkpoint protein BUB3 (Jiang et al., 2014). These mRNAs and the other RNAs communicating with them may regulate the contraction and metabolism of the myometrium during labor, though further investigation is still needed.

Postpartum hemorrhage, a complicated multifactorial process, remains a leading cause of maternal morbidity and mortality. The common causes of excessive bleeding are uterine atony (70%), retained placenta, genital tract injuries, and coagulopathy (Oyelese et al., 2010). According to the results of our transcriptome profiles in different amounts of bleeding after delivery, a number of differentially expressed RNAs were identified. Most of these mRNAs were positively correlated with bleeding volume and markedly enriched functions and pathways of coagulation, inflammatory response, and blood vessel endothelial cell proliferation. Inflammation and wound healing are closely related to the vascular endothelium, and vascular endothelial growth factor is a key factor that regulates this process (Stanley et al., 2015). Our results showed a positive correlation between inflammatory response and blood vessel endothelial cell alteration and the amount of postpartum blood loss. Eight genes were associated with hemostatic pathways (SELL, CAPZB, SLC16A3, PDPN, TNFRSF10B, SH2B2, NFE2, and TNFRSF10D), which provided novel insights for the prevention and management of postpartum hemorrhage. Case–control studies including patients with postpartum hemorrhage are needed to confirm our findings. The mechanism through which these genes regulate hemostasis in labor requires further studies in animal experiments.

There are several limitations in this study that should be noted. Due to the difficulty of myometrium tissue collection, the transcriptome data were derived from a limited number of samples, and these myometrium variables (labor duration and blood loss) were not evenly distributed for each time point. The study will benefit from validation of results with larger sample cohorts, which requires further research. In this study, six participants underwent failure to progress. Failure to progress was due to many reasons. In our study, the cases of “failure to progress” were all clinically considered to be caused by cephalopelvic disproportion instead of primary uterine atony. The assessment of the labor start time primarily relied on routine clinical method (determined using cardiotocography and by digital examination after self-reporting regular contractions); therefore, it was nearly impossible to record the precise time point when the labor started, even though all the participants were hospitalized before labor started. Similarly, blood loss was estimated using routine measurement methods, which were a mix of uterine bleeding from caesarean incision and postoperative vaginal bleeding. In addition, transcriptome data were derived from total RNA of the whole myometrial tissues, and we were unable to determine which type of cell in the human myometrium contributed to the significant changes in gene expression identified from its data sets. Single-cell omics can provide a better investigation of the cell-specific changes in the myometrium, which could help to identify appropriate targets for future clinical interventions.

By utilizing RNA-seq with advanced bioinformatics techniques, we have shown that there were significant changes in the transcription levels in the myometrium at different phases of labor. Then we analyzed the functions and pathway alterations and constructed a regulatory network of parturition. Our study presented a method of participants’ selection criteria of labor duration and cervical dilation status. Transcriptome and its ceRNA network correlated with labor duration and blood loss provided certain potential key RNAs for subsequent molecular mechanism research, which could help determine the causes of changes in human myometrium function during physiological and pathological labor.

## Data Availability

The datasets presented in this study can be found in Genome Sequence Archive (GSA) repository with accession number PRJCA009585.
