# Supplementary figures and images for: Time Course Analysis of Transcriptome in Human Myometrium Depending on Labor Duration and Correlating With Postpartum Blood Loss
Source: Front Genet. 2022 Jun 28;13:812105. doi: 10.3389/fgene.2022.812105 (PMC9273953; doi:10.3389/fgene.2022.812105)

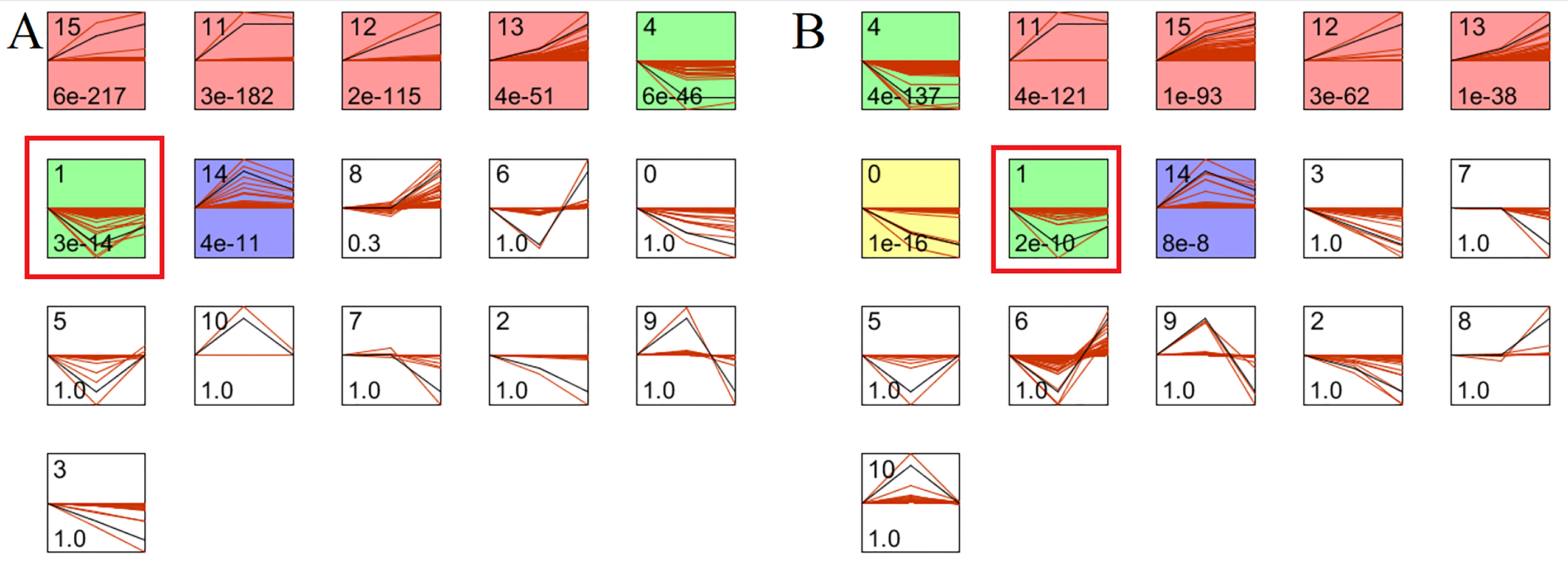

Supplement: Supplementary file 1 [file Image1.jpg]
